# Pharmacological Inhibition of mTORC2 Reduces Migration and Metastasis in Melanoma

**DOI:** 10.3390/ijms22010030

**Published:** 2020-12-22

**Authors:** Jessica Guenzle, Harue Akasaka, Katharina Joechle, Wilfried Reichardt, Aina Venkatasamy, Jens Hoeppner, Claus Hellerbrand, Stefan Fichtner-Feigl, Sven A. Lang

**Affiliations:** 1 Department of General and Visceral Surgery, Medical Center-University of Freiburg, Faculty of Medicine, University of Freiburg, Hugstetter Strasse 55, 79106 Freiburg, Germany; jessica.guenzle@uniklinik-freiburg.de (J.G.); o.harue.0277@gmail.com (H.A.); katharina.joechle@uniklinik-freiburg.de (K.J.); jens.hoeppner@uniklinik-freiburg.de (J.H.); stefan.fichtner@uniklinik-freiburg.de (S.F.-F.); 2 German Cancer Consortium (DKTK), 69120 Heidelberg, Germany; wilfried.reichardt@uniklinik-freiburg.de; 3 German Cancer Research Center (DKFZ), 69120 Heidelberg, Germany; 4 Department of Radiology Medical Physics, Medical Center-University of Freiburg, Faculty of Medicine, University of Freiburg, Killianstrasse 5a, 79106 Freiburg, Germany; aina.vnkt@gmail.com; 5 Service de Radiologie 1, Hôpital de Hautepierre, Hôpitaux Universitaires de Strasbourg, 1 Avenue Molière, 67098 Strasbourg, France; 6 Laboratory Stress Response and Innovative Therapies “Streinth”, Inserm IRFAC UMR_S1113, Université de Strasbourg, 67098 Strasbourg, France; 7 Institute of Biochemistry, Friedrich–Alexander University Erlangen-Nürnberg, Fahrstrasse 17, 91054 Erlangen, Germany; claus.hellerbrand@fau.de; 8 Comprehensive Cancer Center Freiburg-CCCF, Medical Center-University of Freiburg, 79106 Freiburg, Germany; 9 Department of Surgery and Transplantation, University Hospital RWTH, 52074 Aachen, Germany

**Keywords:** mTORC2, melanoma, migration, metastasis

## Abstract

Despite recent advances in therapy, liver metastasis from melanoma is still associated with poor prognosis. Although targeting the mTOR signaling pathway exerts potent anti-tumor activity, little is known about specific mTORC2 inhibition regarding liver metastasis. Using the novel mTORC2 specific inhibitor JR-AB2-011, we show significantly reduced migration and invasion capacity by impaired activation of MMP2 in melanoma cells. In addition, blockade of mTORC2 induces cell death by non-apoptotic pathways and reduces tumor cell proliferation rate dose-dependently. Furthermore, a significant reduction of liver metastasis was detected in a syngeneic murine metastasis model upon therapy with JR-AB2-011 as determined by in vivo imaging and necropsy. Hence, our study for the first time highlights the impact of the pharmacological blockade of mTORC2 as a potent novel anti-cancer approach for liver metastasis from melanoma.

## 1. Introduction

Metastasis is the primary cause of death in most cancer patients [1]. Although novel therapeutic approaches have been developed, the clinical outcome of patients with melanoma is poor and dependant on the presence of metastasis [2]. A common site of melanoma metastasis is the liver, with limited treatment options regarding surgery and systemic chemotherapy [3]. Even though novel therapeutic options such as immune checkpoint inhibitors or targeted therapy of BRAF^V600^ mutation are available, overall survival is still limited upon the development of liver metastasis [4,5]. Hence, new insights into the signaling pathways and mechanisms promoting liver metastasis are needed.

The steps of metastasis are dependent on motility and migration processes and are similar to non-neoplastic cells during physiological processes like angiogenesis, homing, morphogenesis, and embryonic development [6]. Physiological invasion is tightly regulated and ends when the stimulus diminishes; the circulation and invasive formation of detached cells from a tumor result from an imbalance of regular signaling [6,7]. Furthermore, the investigations of Sarna et al. showed that the content of melanin in melanoma cells can influence the ability of tumor cells to spread by changes in mechanical elasticity [8,9]. In addition, the extracellular matrix constitutes a boundary for tumor cells, and the expression level of matrix metalloproteinases (MMP) is correlated with tumor cell invasiveness [10,11]. Among the family of MMPs, MMP2 is known to be a key player in metastasis development [12]. Interestingly, MMP2 has been shown to be regulated at least in part via mTORC2/Akt signaling [13,14]. With regards to liver metastasis, we have previously pointed out the significance of mTORC2/Rictor in melanoma and pancreatic cancer models by using RNA interference [15]. In the meantime, a pharmacological inhibitor has been developed with potential clinical application, extending our previous work. The mammalian target of rapamycin (mTOR) is a serine/threonine kinase that forms two multiprotein complexes, mTORC1 and mTORC2, with distinct subunits [16]. While mTORC1 is extensively studied due to the availability of pharmacological mTORC1 inhibitors (e.g., rapamycin), the role of mTORC2 in cancer is less clear. Nonetheless, the major regulatory subunit of mTORC2, Rictor, is overexpressed in many cancers and involved in certain cancer-associated processes such as therapy resistance [17,18]. Moreover, mTORC2 plays an important role in the promotion of cancer cell survival, proliferation, growth, and motility via activation/phosphorylation of Akt^Ser473^, a key regulator of the insulin/PI3K pathway [19]. Finally, the constitutive hyperactivation of Akt in many cancers leads to an intense feedback activation of mTORC2 [20]. Taken together, inhibition of mTORC2 seems to be a valuable target for anti-cancer therapy.

Over the last decade, several attempts were made to address this issue. Although PI3K/Akt inhibitors displayed a promising response in preclinical and early phase clinical assessment, the high toxicity profile, and development of resistance limits the use of these agents [21]. In addition, it is well established that mTORC1 inhibitors (rapalogs) often lead to feedback activation of PI3K/Akt via mTORC2 [22,23]. Hence, dual kinase inhibitors for PI3K signaling and the rapamycin-sensitive mTORC1 pathway, as well as ATP-competitive inhibitors of mTORC1/2, were assessed in preclinical and clinical settings with limited efficacy [24,25,26]. However, selective inhibition of mTORC2/Rictor in cancer is commonly evaluated using RNAi approaches due to the lack of specific inhibitors so far [15,27,28]. Interestingly, recent work from Benavides–Serrato and coworkers described for the first time the use of a specific pharmacological mTORC2 inhibitor (JR-AB2-011) that was identified from a NCI/DTP small molecule compound library in an experimental glioblastoma model [29].

The aim of the present study was to investigate the effect of the specific mTORC2 blockade by JR-AB2-011 on the migration and invasion capacity of melanoma cells as well as the formation of liver metastasis and to explore the possible molecular pathways in vitro and in vivo.

## 2. Results

### 2.1. Inhibition of mTORC2 Reduces Cell Viability and Proliferation of Melanoma Cells

**Figure 1:**
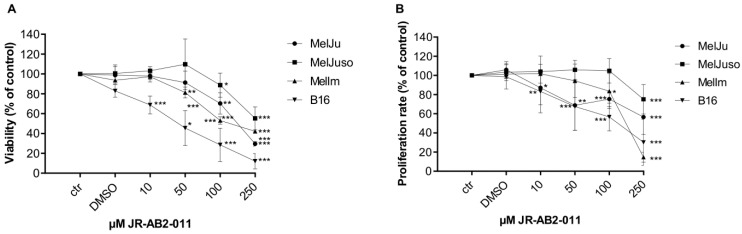
Inhibition of mTORC2 reduced cell viability and proliferation dose-dependently. (**A**) The dose-dependent toxic effect of JR-AB2-011 (10–250 µM) was assessed by 3-(4,5-Dimethylthiazol-2-yl)-2,5-diphenyltetrazoliumbromid (MTT) assay over 48 h in human and murine melanoma cells. Viability was plotted relative to untreated controls set to 100% (± standard deviation (SD) of three independent experiments). (**B**) bromdesoxyuridin BrdU incorporation assay indicates relevant inhibition of proliferation in human and murine melanoma cells by JR-AB2-011 (10–250 µM) after 48 h compared with the loss of cells in MTT assay. Treatment was normalized to untreated controls (± SD of three independent experiments). Asterisks (* *p* ≤ 0.05, ** *p* ≤ 0.01, *** *p* ≤ 0.001) indicate significance between treatment and the control.

To investigate the influence of the pharmacological inhibition of mTORC2 by JR-AB2-011 on cell viability and proliferation of different melanoma cell lines, we performed 3-(4,5-Dimethylthiazol-2-yl)-2,5-diphenyltetrazoliumbromid (MTT) and bromdesoxyuridin (BrdU) incorporation assays. Cell viability of all human melanoma cell lines was significantly reduced in a dose-dependent manner after exposure to 100 µM JR-AB2-011 for 48 h (MelJu, 70%, *p* = 0.017; MelJuso, 88%, *p* = 0.1; MelIm, 52%, *p* < 0.001) whereas murine melanoma cells were significantly affected by 10 µM JR-AB2-011 (B16, 63%, *p* = 0.04) (Figure 1A). Maximum reduction of cell viability at 250 µM after 48 h was observed for human melanoma cells at 29% (MelJu *p* < 0.001), 55% (MelJuso *p* = 0.0001), 42% (MelIm *p* < 0.001) and for murine melanoma cells at 17% (B16 *p* < 0.001). Investigation of the cell viability after 24 h exposure to JR-AB2-011 showed a significant reduction starting at 100 µM or 250 µM for melanoma cells (*p* < 0.001) (Figure S1A). Reduction of cell viability is slightly strengthened after 72 h compared to 48 h (*p* < 0.001) (Figure S1B).

Regarding proliferation in sub-toxic concentrations, melanoma cells exposed to the mTORC2 inhibitor for 48 h showed a reduced rate of proliferation of up to 14% (MelIm, *p* < 0.001) at the maximum concentration of 250 µM JR-AB2-011 (Figure 1B). At 100 µM JR-AB2-011, we observed a moderate reduction of the cell proliferation to 83% for the melanoma cell lines. Exposure to JR-AB2-011 for 24 h led to a similar reduction in the proliferation rate of melanoma compared to 48 h (Figure S1C).

In summary, exposure of melanoma cells to JR-AB2-011 led to a significantly reduced cell viability and a slight reduction in cell proliferation.

### 2.2. Exposure to JR-AB2-011 Leads to Impaired Activation of Akt Signaling

**Figure 2:**
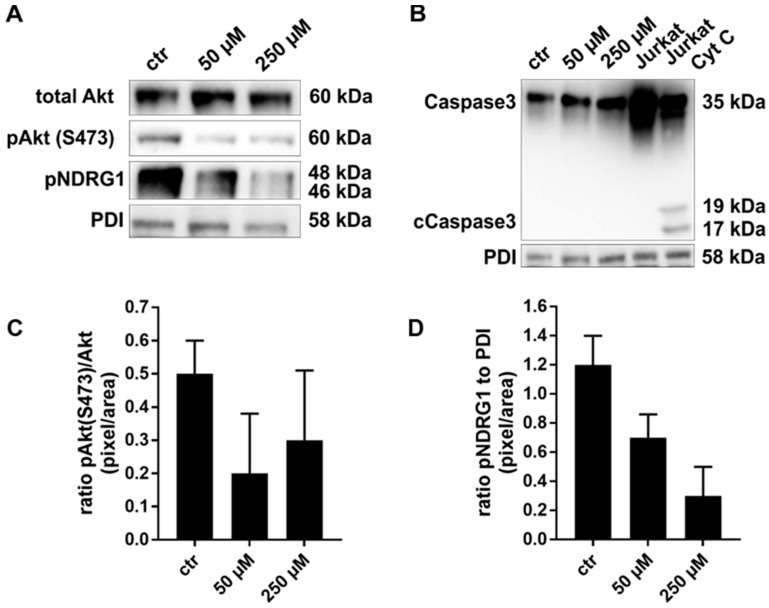
JR-AB2-011 addressed the mTORC2 specific Akt and NDRG1 signaling. Representative Western Blot analysis of total Akt (60 kDa), pAkt^Ser473^ (60 kDa), and pNDRG1 (58 kDa) in human melanoma (MelIm) (**A**) after treatment with 50 µM and 250 µM JR-AB2-011 indicates a dose-dependent reduction in anti-apoptotic Akt signaling in melanoma cells. Representative Western Blot analysis of Caspase 3 (35 kDa) and activated Caspase 3 (cCaspase 3, 17 kDa) indicates no influence of JR-AB2-011 on classical apoptosis in melanoma cells (MelIm) (**B**). Untreated as well as cytochrome c–treated (Cyt c) Jurkat cells were used as negative and positive controls. Densitometry reveals up to 50% reduction of pAkt^Ser473^ phosphorylation as determined by ratio of pAkt^Ser473^/Akt (**C**). Further, phosphorylation of NDRG1 was dose-dependently affected in melanoma cells. Densitometry reveals up to a 62% reduction of NDRG1 phosphorylation (**D**). 20 μg of protein lysates were separated by SDS-PAGE in three independent experiments, displayed are cropped blots; for full gels see Supplementary Data. PDI was used as a loading control.

Akt is a modulator of anti-apoptotic signaling that is mainly regulated via phosphorylation at serine 473 by mTORC2. Therefore, the inhibition Akt phosphorylation after pharmacological mTORC2 blockade was investigated by Western Blot analysis. Concentrations lower than 50 µM did not affect the phosphorylation of Akt (Figure S2). Therefore, 50 µM and 250 µM were chosen as representatives for low and high concentrations, as discussed below. Treatment of MelIm cells with 50 µM and 250 µM JR-AB2-011 for 48 h led to an inhibition of Akt phosphorylation and a dose-dependent decrease in NDRG1 activation (Figure 2A,C,D). Since NDRG1 is a physiological substrate of SGK1 and is not regulated by mTORC1 but only by mTORC2, we demonstrate the specific targeting of mTORC2 after 48 h. Similar results were obtained for all melanoma cell lines (data not shown). Since classical apoptosis can be regulated by anti-apoptotic Akt signaling, we subsequently addressed this issue. To exclude the involvement of caspase-dependent apoptosis, we analyzed the cleavage of caspase 3 (cCaspase 3) by Western Blot analysis (Figure 2B). Cytochrome c treated Jurkat cells (Jurkat Cyt C) revealed the activation of caspase 3 compared to non-treated Jurkat cells (Figure 2B). However, melanoma cells (MelIm) exhibited no cleavage and activation of caspase 3 after 48 h of exposure to 50 µM and 250 µM JR-AB2-011. In summary, our results indicate that inhibition of mTORC2 impairs the pAkt^Ser473^ and NDRG1 signaling as well as classical apoptosis.

### 2.3. mTORC2 Blockade Inhibits Migration and Invasion Capacity by Regulation of MMP2

**Figure 3:**
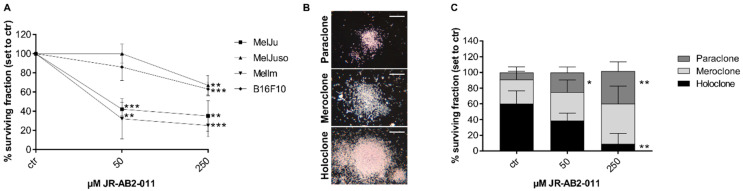
Inhibition of mTORC2 reduced surviving fraction and colony-forming ability. Treatment with JR-AB2-011 (50–250 µM) for 48 h decreased the colony-forming ability significantly in melanoma cells over two weeks in a dose-dependent manner (*p* < 0.002) (**A**). Representative images (**B**) show the characteristic morphology of the para-, mero- and holoclones of MelJu. The morphology of the colonies of MelJu altered significantly from the large and rapidly growing holoclone to more miscellaneous meroclones and the small and terminal paraclones in a dose-dependent manner (*p* < 0.04) (**C**). Asterisks (* *p* ≤ 0.05, ** *p* ≤ 0.01, *** *p* ≤ 0.001) indicate significance between treatment and control. Scale bar indicates 500 µm.

The cell surviving fraction (SF) points to a cell survival curve that is dependant on the dose of the specific mTORC2 inhibitor. Two of three human melanoma cell lines (MelJu, MelIm) exposed to 50 µM revealed a SF less than 50% compared to control (*p* = 0.0001) (Figure 3A). After exposure to 250 µM, all melanoma cell lines demonstrated significantly reduced SF after 48 h (*p* < 0.002). As described by Barrandon and Green, colonies of melanoma cells exposed to JR-AB2-011 were subdivided into holoclone-, paraclone- and meroclone-like colonies based on their morphology [30] (Figure 3B). Subdivided fractions of non-treated melanoma cells (MelJu) revealed 60% holoclone-like cells exhibiting differentiated cells with high growth potential in cell-rich colonies (Figure 3C). Exposed to 50 µM JR-AB2-011, the cells varied to more meroclone-like cells in more loosened cell colonies with lower growth potential, whereas the paraclone-like colonies increased significantly (*p* = 0.04). Treatment with 250 µM JR-AB2-011 led to a significantly reduced number of holoclone-like colonies 2(*p* = 0.01) and concurrently to a significant increase of the paraclone-like colonies (*p* = 0.01) with a differing capacity of growth. Melanoma cell lines MelJuso (Figure S3A,D), MelIm (Figure S3B,E), and B16 (Figure S3C,F) obtained similar results in the reduction of the holoclone-like colonies and the increase of meroclone-like and paraclone-like colonies (Figure S3A–F).

**Figure 4:**
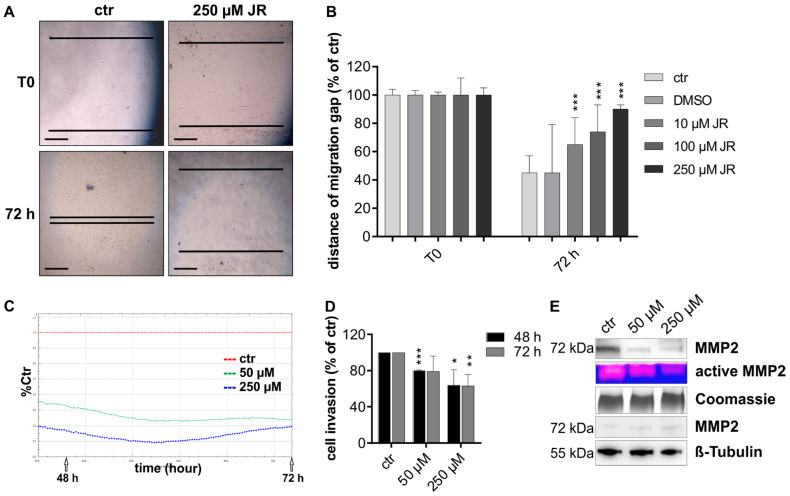
mTORC2 blockade inhibited migration and invasion capacity by regulation of MMP2. Migration capacity of MelJu was investigated after exposure of 10–250 µM JR-AB2-011 by wound closure assay over 72 h. Representative images (**A**) show the initial gap (T0) and the gap after 72 h of control and treatment with 250 µM JR-AB2-011. The initial width of the migration gap was 2 mm and is shown as the distance of the migration gap. Scale bar indicates 500 µm. (**B**) Treated cells were normalized to T0 (start of the experiment) set to 100% (±SD of three independent experiments). Asterisks (* *p* ≤ 0.05, ** *p* ≤ 0.01, *** *p* ≤ 0.001) indicate significance (10µM *p* = 0.000017; 100µM *p* = 0.000004; 250µM *p* = 0.000001) between treatment and the control (72 h). Real-time invasion of MelJuSo (**C**) was investigated with RTCA measurement over 72 h after exposure to JR-AB2-011 (50–250 µM). Arrows indicate the time points of 48 h and 72 h. The red line shows untreated control set to 100% compared to treatment with 50 µM (green) and 250 µM (blue). All cells started at the same time point. The treatment with JR-AB2-011 significantly reduced the invasion capacity of MelJuSo after 48 h and 72 h (**D**). Asterisks (* *p* ≤ 0.05, ** *p* ≤ 0.01, *** *p* ≤ 0.001) indicate significance between treatment and the control set to 100%. Representative images (**E**) of Western Blot analysis and zymography analysis with conditioned media of MelJuSo (upper panel) indicate a reduction of expression and activation of MMP2 in melanoma cells, whereas less inactive MMP2 accumulated in the cell lysate (lower panel) when treated with 50–250 µM JR-AB2-011. 4 µg of protein of the supernatant and 20 µg of the cell lysates were screened for MMP2. Coomassie staining of the gel was used as a loading control for conditioned media, β-Tubulin was used as a loading control for cell lysates. Figures are representative for three independent experiments, displayed are cropped blots; for full gels, see Supplementary Data.

To investigate the migration capacity of the melanoma cells, we performed a wound closure assay over 72 h. The initial migration gap of 2 mm is shown for untreated and treated melanoma cells in Figure 4A. The migration gap was nearly closed by untreated melanoma cell (MelJu) after 72 h whereas cells exposed to 250 µM JR-AB2-011 revealed no relevant migration capacity into the wound (Figure 4A). The ability to close the gap is dose-dependently reduced by inhibition of mTORC2 compared to untreated controls, as shown by the remaining distance of the migration gap (Figure 4B). To further investigate the infiltrative capacity, we performed electronic real-time cell analysis (RTCA) measurements (Figure 4C). Quantitative monitoring of transwell experiments over 72 h revealed a significant dose-dependent reduction of the invasive capacity of melanoma cells (MelJuso, *p* < 0.001) exposed to 50 µM and 250 µM JR-AB2-011 compared to untreated controls (Figure 4D). Similar results for transwell invasion were obtained in all melanoma cell lines for 48 h and 72 h (Figure S4A = MelJu; 3B = MelIm; 3C = B16). Upregulation of pro-invasive enzymes such as matrix metalloproteinase-2 (MMP2) contributes to the invasion of tumor cells. Since the zymogen MMP2 is activated in the extracellular matrix, we investigated the protein and activation levels in the supernatant of the cell culture layer. The supernatant of cells exposed to 50 µM and 250 µM of JR-AB2-011 contained a considerable reduction of MMP2 protein in a denaturing Western Blot analysis compared to controls (MelJuso, Figure 4E). Likewise, zymography analysis of the conditioned media of the cells revealed a relevant decrease of active MMP2 in response to mTORC2 inhibition in melanoma cells in non-degrading gelatin gels (Figure 4E). The investigation of the whole cell lysate of exposed cells showed an equal protein level of MMP2 and implicated a malfunction of the export of MMP2. Taken together, the specific inhibition of mTORC2 leads to reduced cell mobility and migration by regulation of MMP2 activity in melanoma cells in vitro.

### 2.4. Pharmacological Inhibition of mTORC2 In Vivo Reduced the Metastasis Load in the Liver

**Figure 5:**
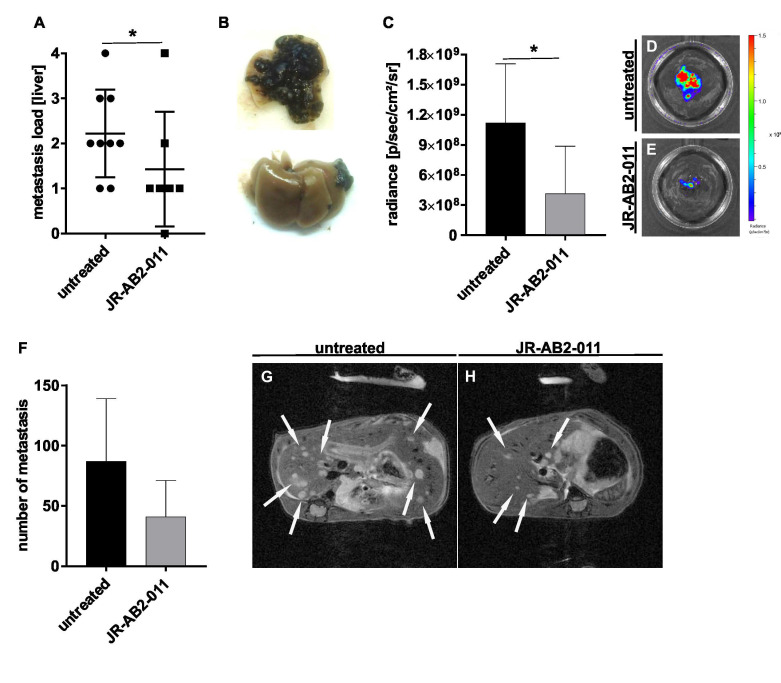
Pharmacological inhibition of mTORC2 reduced the metastasis load in the liver of a syngeneic mouse model. The reduction of the metastasis load in the liver after treatment with JR-AB2-011 was investigated by a mouse model with a splenic injection of B16 murine melanoma cells. The load of total metastasis of untreated controls and treated animals was macroscopically scored after dissection (0 = no tumor load; 1 = very low tumor load; 2 = low tumor load; 3 = medium tumor load; 4 = high tumor load) and indicates a significant reduction of total metastasis (*p* = 0.01) (*n* = 16) (**A**). Representative images (**B**) show the characteristic of classification type 4 (up) and type 1 (down). Due to the use of luciferase-marked B16 cells, the radiance of the metastasis in the murine liver was measured by bioluminescence imaging and found statistically significant (*p* = 0.01) (*n* = 16) (**C**). Asterisks (* *p* ≤ 0.05) indicate significance between treatment and untreated control. Representative images of BLI measurement show the radiance of the dissected liver of untreated (**D**) and treated mice (**E**). The number of single metastasis in the liver was assessed and quantified by MRI measurement with 30 slices, covering the whole liver (*p* = 0.25) (*n* = 6; 3/group) (**F**). White arrows in representative images of MRI show exemplary metastasis of untreated (**G**) and treated mice (**H**).

To confirm the results with regards to inhibition of metastases formation in vivo, we used the syngeneic murine splenic injection model with B16 melanoma cells as described [15]. Mice were daily treated with 20 mg/kg BW JR-AB2-011 or solvent starting one day after inoculation of tumor cells by the end of the experiment after 13 days. No side-effects such as weight loss were observed in mice upon treatment with the inhibitor (Figure S5H). In vivo assessment of metastasis load was performed by Magnetic Resonance Imaging (MRI) and Bioluminescence Imaging (BLI) measurements at different time points. Upon necropsy (*n* = 16;8/group), metastasis load was assessed by three independent investigator and scored (Figure 5B; very high metastasis = 4; very low metastasis = 1; no metastasis = 0). The analysis revealed that animals treated with the mTORC2 inhibitor showed significantly decreased hepatic tumor burden after dissection. The majority of animals in the treatment group showed a score of 1 (*n* = 4), whereas, in the control group, the main score was 2 (*n* = 4) (*p* = 0.01) (Figure 5A). In one animal, no hepatic tumor burden was detectable after treatment with JR-AB2-011. BLI imaging of dissected murine liver tissue confirmed the assumption of the macroscopic score in mice treated with the mTORC2 inhibitor. In the treatment group, a significant reduction of the radiance emitted by the transfected melanoma cells was measured, indicating less hepatic tumor burden as shown by representative images (*p* = 0.01) (Figure 5C–E). Images of ex vivo measurement for all mice are provided in Figure S5G. MRI indicated 53% less single metastasis in the treatment group, measured in three mice per group at day 13, although this difference did not reach the level of significance due to the limited number of mice that underwent MRI (*p* = 0.25) (Figure 5F). Single tumors in the liver appeared smaller in size compared to untreated controls (Figure 5G,H). The total volume of the primary spleen tumor in MRI was calculated 49% less in treated mice compared to controls (Figure S5A–C). Similar, BLI measurement of the whole body of living mice displayed 47% less total melanoma cells upon JR-AB2-011 therapy (Figure S5D–F).

**Figure 6:**
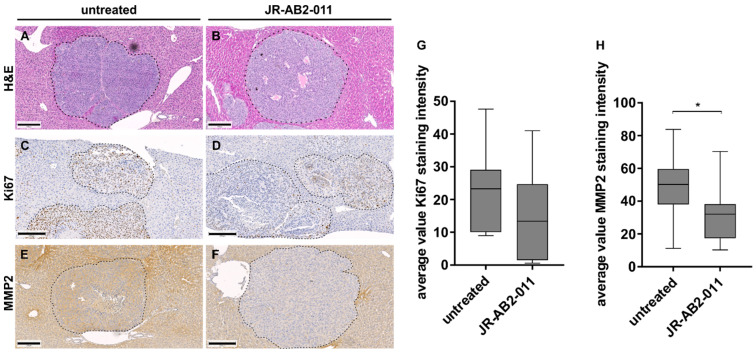
In vivo inhibition of mTORC2 impaired metastasis load as well as proliferation and MMP2 expression in the liver. A reduction of the metastasis load could be observed by hematoxylin and eosin (H and E) staining and is shown for untreated (**A**) and treated (**B**) mice by representative images of the dissected murine liver. The impact of the treatment on cell proliferation (Ki67) was analyzed with the ImageJ immunohistochemistry (IHC) toolbox and plotted as average value Ki67 staining intensity (*n* = 16; 8/group) (**G**). Representative images of immunohistochemistry of untreated mice (**C**) and treated mice (**D**) indicate a decreased number of proliferating cells within the metastasis in the liver tissue. Staining of MMP2 revealed a significant decrease in treated mice (**F**) compared to controls (**E**), analyzed with ImageJ IHC toolbox and plotted as average value MMP2 staining intensity (*n* = 16) (**H**). Metastases are marked with a dashed line. Asterisks (* *p* ≤ 0.05) indicate significance between treatment and the control. Scale bar indicates 200 μm (magnification × 10).

On histopathological assessment with H and E staining, we identified single metastasis in the liver tissue (Figure 6A,B). The proliferation rate of tumor cells in the metastases was determined using Ki67 staining (Figure 6C,D). The average value of the Ki67 staining intensity revealed a decrease of proliferation in the treatment group but did not reach statistical significance (Figure 6G). Since active MMP2 was reduced in the conditioned media of melanoma cells, we also investigated the MMP2 expression immunohistochemically (Figure 6E,F). Results revealed a significant reduction of MMP2 staining intensity in liver metastases upon treatment with JR-AB2-011 compared to controls (*p* = 0.03) (Figure 6H).

From these results, we conclude that the pharmacological inhibition of mTORC2 with JR-AB2-011 reduced the formation of liver metastasis of melanoma cells in vivo.

## 3. Discussion

Liver metastasis in melanoma is associated with a poor prognosis due to rapid systemic dissemination of the disease and limited therapeutic options [31]. Even though novel systemic therapies are available, the 3-year overall survival upon detection of liver metastases is less than 24% [32]. Therefore, new insights and targets based on molecular mechanisms involved in metastasis are urgently needed.

The role of mTORC2 in cancer is increasingly studied [33,34,35]. However, the assessment of mTORC2 inhibition has so far been difficult due to the lack of specific inhibitors. mTOR inhibitors such as rapamycin are known to block mTORC1 signaling in most cells. However, treatment of tumor cells with these agents can also lead to activation of Akt, the most important mTORC2 downstream target, via phosphorylation of Akt at serin 473, in a mTORC2/Rictor dependent manner [36,37]. The obvious concern regarding this Akt activation is a pro-tumorigenic effect due to the central role of Akt in cancer. Furthermore, dual kinase inhibitors not only affect mTORC2 but also mTORC1, providing a less clear picture of the specific effects of mTORC2 inhibition. Recently, Benavides-Serrato et al. described a novel specific mTORC2 inhibitor, JR-AB2-011, which might help to overcome this issue [29]. In this study, treatment with JR-AB2-011 in a range of 0.5–10 µM impaired mTORC2 activation as determined by inhibition of mTORC2 downstream targets in vitro and tumor growth in vivo in a glioblastoma model [29]. In contrast, we needed a higher dosing of the same inhibitor in our study on melanoma cells to obtain similar effects. Considering the literature, this impact may not be astonishing. Treatment with mTOR(C1) inhibitors such as rapamycin or the dual-kinase inhibitor OSI027 reveal similar findings when comparing the effects in glioblastoma and visceral tumors with higher dosing needed for non-CNS tumors [38,39,40,41,42]. However, using JR-AB-011, we could show an impairment of mTORC2 specific Akt phosphorylation at serine 473 in melanoma cells starting at 50 µM. This is of particular importance since Akt is implicated in cancer due to its crucial role in the inhibition of apoptosis and cancer cell survival [43]. In addition, treatment with JR-AB2-011 led to decreased activation of NDRG1, which is another specific target of mTORC2 [44,45]. Some studies revealed that NDRG1 is highly expressed in hepatocellular carcinoma and cervical cancer and interrupts many metastasis-associated processes, including epithelial-mesenchymal transition (EMT) [46]. However, silencing of NDRG1 led to reduced proliferation and invasion capacity as well as tumor growth in vitro and in vivo in these studies [46,47]. Taken together, we show that treatment with JR-AB2-011 impairs the mTORC2-specific targets Akt and NDRG1 and, therefore, may be a modulator of cancer cell survival, motility, and tumor progression.

Next, we assessed the mechanism that connects mTORC2 blockade with JR-AB2-011 to the inhibition of metastatic processes. Besides the impairment of cancer cell motility, our results showed a significant decrease of active MMP2 in the conditioned media of the melanoma cells in vitro. Moreover, we inevitably detected a significant decrease of MMP2 in the hepatic metastasis in vivo. Matrix metalloproteinases are known to drive invasion and migration processes in vivo by degradation of the extracellular matrix (ECM) [48,49]. In our experiments, the intracellular MMP2 protein expression in the cell lysate is not affected by mTORC2 inhibition, although active MMP2 was considerably downregulated by treatment with JR-AB2-011. We, therefore, assume that the principle of regulation by the mTORC2 signaling must occur while transmembrane transport and activation of MMP2. Only a few studies have analyzed a connection of MMP2 and mTORC2/Akt. Liang et al. recently found that Rictor regulates the vasculogenic mimicry of tumor cells via the activation of MMP2 by the PI3K/Akt signaling [14]. In addition, MMP2 is also known to serve as an important downstream effector of PI3K/Akt signaling in breast cancer [50]. In line with these reports, the current study adds further evidence to the fact that there is a functional bridge between mTORC2, Akt, and MMP2. In summary, we provide evidence that the pharmacological inhibition of mTORC2 may act as a promising approach to affect one of the most important signaling pathways for cell invasion and metastasis.

Finally, we evaluated our results regarding the mTORC2 inhibition with JR-AB2-011 using a syngeneic mouse model in vivo. First, we found a trend towards reduced tumor cell proliferation in the liver metastasis in vivo (as determined by Ki67 staining), which is in line with our in vitro results regarding the impairment of tumor cell viability. Second, we detect a significantly reduced expression of MMP2, as mentioned above. Third, and most importantly, we observed a significant reduction of the total metastasis load in the liver by MRI, BLI, and visual observation. To the best of our knowledge, this is the first time that mTORC2 blockade with JR-AB2-011 was assessed in an in vivo model for liver metastasis. Indeed, the impact of Rictor blockade on liver metastasis has been evaluated by others and us before. Sun and coworker found no liver metastases upon RNAi-mediated Rictor inhibition in a xenograft model of renal cell carcinoma [27]. We described reduced hepatic metastasis burden in melanoma and pancreatic cancer models using si-/sh-RNA approach for targeting Rictor [15,28]. Nonetheless, the use of JR-AB2-011 as a specific pharmacological inhibitor for mTORC2 has not been described in this context so far. Moreover, the current study uses for the first time JR-AB2-011 in a syngeneic mouse model in vivo. In general, the use of a syngeneic model has some potential advantages, including the presence of a functional immune system and the existence of a proper microenvironment. In metastasis research, the splenic injection model is commonly used and established, although it harbors some potential limitations [51,52]. Particular critics are its inability to represent the entire metastatic process but only the late stage of the metastatic cascade due to the missing of a primary tumor [51]. In our experiments, we found that no liver metastasis occur when no primary tumor was formed. In addition, liver metastases were found just after a minimum of three days upon primary tumor detection (data not shown). This observation might at least in part overcome the mentioned critics. Taken together, results from our in vivo model confirm the observations from the in vitro experiments regarding the beneficial effect of mTORC2 inhibition on liver metastasis.

In conclusion, the current study shows that treatment with JR-AB2-011 substantially impairs metastasis via inhibition of mTORC2 dependent mechanisms. Hence, we provide further evidence that mTORC2 is a promising novel target for potent novel anti-cancer therapies in metastasizing cancers.

## 4. Materials and Methods

### 4.1. Cell Culture

The human melanoma cell lines MelJu, MelJuSo, and MelIm, as well as the murine cell line B16F10 (ATCC, VA, USA), were cultured in RPMI containing 10% fetal bovine serum at 37 °C in a humidified atmosphere containing 5% CO_2_. The pharmacological mTORC2-inhibitor JR-AB2-011 was obtained from Aobious, Gloucester, MA, USA, and dissolved in DMSO. Cells were exposed to JR-AB2-011 concentrations between 10 and 250 µM for up to 72 h. The final concentration of DMSO was not higher than 0.2% (when using 250 µM JR-AB2-011).

### 4.2. Viability Assay

Cell viability was determined by MTT Assay. 5 x 10^4^ cells were grown in 96 well plates with complete RPMI and treated with 10-250 µM JR-AB2-011 for up to 72 h. MTT Assay was performed as described before [53]. Absorbance at 570 nm was measured (Tecan, Männedorf, Switzerland). Percentages were calculated relative to the viability of untreated controls set to 100%. The experiment was done in triplicate and is representative of a minimum of 3 independent studies.

### 4.3. Cell Proliferation Assay

Cell proliferation was monitored by 5′-bromodeoxyuridine (BrdU) incorporation assay (Roche Diagnostics, Mannheim, Germany). 5 × 10^4^ cells were seeded in 96 well plates and treated with JR-AB2-011 up to 250 µM. Cells were stained with BrdU following the manufacturer’s instructions. The percentage of cells exhibiting genomic BrdU incorporation was measured by absorbance at 370 nm (Tecan, Männedorf, Switzerland). Percentages were calculated relative to the proliferation of untreated controls set to 100%.

### 4.4. Migration and Invasion Assay

Cells were seeded in 24 well plates with silicon bars to create a gap of about 2 mm as described before and treated with JR-AB2-011 up to 250 µM for 72 h [10]. The time required for wound closure was measured by microscopy (Leica DMIL LED, Camera Leica DFC450C, Wetzlar, Germany) and documented up to 72 h. Electronic RCTA analysis for tumour invasion was performed with 4 × 10^4^ serum-starved melanoma cells over 72 h. Cells were resuspended in 50, 100, and 250 µM JR-AB2-011 in serum-free medium and added to the upper chamber of equilibrated xCELLigence CIM-plates (OLS Omni Life Science, Bremen, Germany). Values for the invasion of the cells were detected every 15 min for 72 h. RCTA Data Analysis Software 1.0 was used for the presentation of the curves and calculation of the slopes at 48 and 72 h.

### 4.5. Colony Forming Unit Assay

Cells were seeded in flasks and exposed to up to 250 µM JR-AB2-011for 48 h. Five hundred cells were reseeded in six-well plates and cultured in complete RPMI. After two weeks, cells were washed with PBS, fixed in PFA for 30 min, and stained with hematoxylin for 30 min. Plates were washed with water and air-dried. Counting of colonies was performed by microscopy (Leica DMIL LED, Camera Leica DFC450C, Wetzlar, Germany) and differed in the colony types holoclone, meroclone, and paraclone [30,54]. Plating efficiency (PE) and Surviving fraction (SF) was calculated and set to control to 100% using following equation: PE = # of colonies formed / # of cells seeded × 100%; SF = # colonies formed after treatment / (# of cells seeded × PE).

### 4.6. Western Blotting

Melanoma cells were cultured in serum-free RPMI. The supernatant of JR-AB2-011 treated cells was collected. Equal amounts of protein (4 µg of supernatant, 20 µg of lysate) were applied on 10% SDS-polyacrylamide gels and electrophoresed (BioRad, Munich, Germany) as described before [10]. Blots were incubated with primary antibodies anti-MMP2 (#4022 1:1000 Cell Signaling Technology, Danvers, MA, USA), anti-Akt (#9272 1:1000 Cell Signaling Technology, Danvers, MA, USA), anti-pAkt^Ser473^ (#9271 1:1000 Cell Signaling Technology, Danvers, MA, USA), anti-pNDRG1 (#3852 1:1000 Cell Signaling Technology, Danvers, MA, USA), anti-Caspase3 (#9665 1:1000 Cell Signaling Technology, Danvers, MA, USA), anti-βTubulin (#86298 1:1000 Cell Signaling Technology, Danvers, MA, USA), anti-PDI (sc-74551 1:1000 Santa Cruz, Dallas, TX, USA). Proteins were visualized by enhanced chemiluminescence (BioRad, Munich, Germany). For loading control, a Coomassie-stained SDS-polyacrylamide gel was used for whole protein in the supernatant, and PDI or β-Tubulin was used for the whole cell lysate. Finally, densitometry was performed using ImageJ. The ratio was calculated to pixel/area of reference PDI. Expression of pAkt^Ser473^ was calculated in relation to total Akt.

### 4.7. Gelatin Zymography

Gelatin is a substrate of MMP2 and can be used for the detection of activity in supernatant [10]. Equal amounts of supernatant (4 µg) used for Western blot were applied under non-reducing conditions on 10% copolymerized gelatin-polyacrylamide gels and electrophoresed (BioRad, Munich, Germany) in running buffer (25 mM Tris, 192 mM glycine, 0.1% SDS). Gels were washed two times in 2.5% Triton-X-100/ddH_2_O for 15 min following incubation in development buffer (50 mM Tris, 5 mM CaCl_2_, 0.02% NaN_3_) for 4 h. Gels were fixed by shaking in methanol:ethanol:acetic-acid (4.5:4.5:1) for 15 min and stained with fixation buffer containing 0.1% Coomassie for 2 h. Gels were incubated in fixation buffer until transparent bands appeared. Gels were visualized by ChemiDoc (BioRad, Munich, Germany). Coomassie staining of total protein in conditioned media was used as a loading control.

### 4.8. In vivo Xenograft Experiments

Institutional guidelines for animal welfare and experimental conduct were followed for all animal experiments, which were approved by the Institutional Animal Care and Use Committee and the Regional Administrative Authority under protocol G17/151. All animals received food and water ad libitum. Six weeks old male C57BL/6N mice (*n* = 20) (Charles River, Sulzfeld, Germany) underwent splenic injection of 2.5 × 10^5^ B16 melanoma cells containing firefly luciferase-expressing plasmid pCHMWS_Luciferase under isoflurane anesthesia and 200 mg/kg metamizole pain treatment. Treatment with 20 mg/kg JR-AB2-011 intraperitoneal (i.p.) or solvent control for 13 days was started one day after tumor inoculation (*n* = 10). Thirteen days after intrasplenic injection, mice were terminated.

### 4.9. Bioluminescence Imaging

Metastasis load of the liver was determined alive under Isoflurane anesthesia and upon necropsy by bioluminescence imaging (BLI) using the IVIS Spectrum (Caliper Lifesciences, Mainz, Germany; [55,56]). For BLI, an aqueous solution of D-Luciferine (Promega, Mannheim, Germany, 150 mg/kg BW) was injected i.p. 10 min before the measurement. Living Image software (Caliper Lifesciences, Mainz, Germany) was used to compute regions of interest (ROI) and integrate the total bioluminescence signal in each ROI. Data were analyzed using average radiance (photons/second/cm^2^/steradian) in the ROIs and normalized to a background signal. Additionally, metastasis load and hepatic tumor burden was estimated by three independent investigators and graduated by macroscopic score (0 = no tumor load; 1 = very low tumor load < 10%, e.g., single metastasis; 2 = low tumor load < 40%, e.g., metastasis only on one side of the liver/one liver lobe; 3 = medium tumor load < 70%, more than one liver lobe affected; 4 = high tumor load > 70%, all lobes affected/confluent tumor mass). Upon necropsy, the liver was dissected, weighed, and processed for further analyses.

### 4.10. MRI Measurement

All animals were imaged on a 9.4 T Bruker (Bruker BioSpin MRI GmbH, Ettlingen, Germany, software ParaVision 6.1) with a dedicated whole-body mouse coil. The mice were anesthetized using Isoflurane (Abbott GmbH, Wiesbaden, Germany; 2% vaporized in oxygen) during the examination, placed headfirst in a supine position in the MRI. The body core temperature of the mice was kept at approximately 37 °C using a water bed heating system (Bruker BioSpin MRI GmbH, Ettlingen, Germany) connected to a circulator (Haake SC100 Immersion Circulators, Thermo Fisher Scientific, Waltham, MA, USA), giving a constant flow of hot water. The depth of anesthesia was monitored by the breathing rate of the animal. For the correct placement of the animal and especially the organ of interest, we first performed a fast gradient-echo localizer and secondly a T2-weighted spin-echo RARE (Rapid Acquisition with Relaxation Enhancement) sequence. This sequence was performed to delineate the tumor and eventual metastasis from the surrounding healthy tissue. The RARE sequence in axial orientation featured a FOV of 28 mm^2^, a matrix size of 280 × 280 pixel^2^, and an in-plane resolution of 100 × 100 µm^2^. The slice thickness was 0.50 mm with 0.30 mm slice spacing to achieve optimal image sets of the whole volume (TR/TEeff/FA: 3000 ms/24 ms/180°). The number of slices was 30. Then sequences that appeared highly relevant in the meta-analysis were performed and optimized for their use at 9.4T.

### 4.11. Immunohistochemistry

For immunohistochemistry, paraffin-embedded sections were deparaffinized in ethanol, and endogenous peroxidase was inactivated in 3% hydrogen peroxide (DAKO REAL Peroxidase Blocking Solution (#2023)). Sections were blocked with 1% BSA/PBS before incubating overnight with anti-mouse Ki67 antibody (Abcam #ab1667 1:600) or anti-mouse MMP2 antibody (Abcam #ab37150 1:800). For hematoxylin and eosin (H and E) staining, sections were incubated for 30 sec in hematoxylin and for 10 min in 1% eosin. Briefly, histological views were digitalized, and metastases were outlined. Quantification of the average value staining intensity was measured with ImageJ Plugin *IHC Toolbox* (ImageJ software, NIH, MD, USA).

### 4.12. Statistical Analysis

All experiments were performed in triplicates. Data are shown as mean ± standard deviation (SD). Data were compared using an unpaired two-tailed student’s t-test for in vitro and in vivo analysis; *p* < 0.05 was considered statistically significant. Asterisks (* *p* ≤ 0.05, ** *p* ≤ 0.01, *** *p* ≤ 0.001) indicate significance.

## Supplementary Materials

Supplementary materials can be found at https://www.mdpi.com/1422-0067/22/1/30/s1; Figure S1: Viability of human and murine melanoma cells was assessed by MTT assay after treatment with JR-AB2-011 (10–250 µM) for 24 h (A) and 72 h (B).; Figure S2: Representative Western Blot analysis of pAkt^Ser473^ (60 kDa) in human melanoma (MelIm) after treatment with DMSO (equivalent to 250 µM JR-AB2-011) and 10 µM JR-AB2-011 indicates no alteration in anti-apoptotic Akt signaling in melanoma cells.; Figure S3: The morphology of the colonies of MelJuSo (A), MelIm (B), and B16 (C) altered significantly from the large and rapidly growing holoclone to more miscellaneous meroclones and the small and terminal paraclones in a dose-dependent manner. Figure S4: Real-time invasion of melanoma cells was investigated with RTCA measurement over 72 h after exposure to JR-AB2-011 (50–250 µM) of MelJu (A), MelIm (B), and B16 (C).; Figure S5: Investigation of MRI measurement (total amount of 30 slices per mouse, tumor volume calculated by MIPAV) revealed less tumor volume of the primary spleen tumor in the treated animal group (*n* = 6; 3/group).

## Abbreviations

BLI	bioluminescence imaging
BrdU	Bromdesoxyuridin
ECM	extracellular matrix
FOXO1/3a	Forkhead box protein O1/3a
H and E	hematoxylin and eosin
IHC	immunohistochemistry
i.p.	intraperitoneal
JR-AB2-011	3-(3,4-Dichloro-phenyl)-1-(4-fluoro-phenyl)-1-(5-methyl-4,5-dihydro-thiazol-2-yl)-urea
MTT	3-(4,5-Dimethylthiazol-2-yl)-2,5-diphenyltetrazoliumbromid
mTOR	mammalian target of rapamycin
mTORC1	mammalian target of rapamycin complex 1
mTORC2	mammalian target of rapamycin complex 2
MMP2	matrix metalloproteinase 2
MRI	magnetic resonance imaging
NDGR1	N-myc downstream regulated 1
PDK1	3-phosphoinositide dependent kinase 1
PI3K	phosphoinositide 3-kinase
RAPTOR	regulatory associated protein of mTOR
RICTOR	rapamycin-insensitive companion of mTOR
ROI	region of interest
S6K	ribosomal protein S6 kinase
Ser	serine
SF	surviving fraction
SGK1	serum/glucocorticoid regulated kinase 1
Thr	threonine

## References

[1] Zhou H., Huang S. (2011). Role of mTOR signaling in tumor cell motility, invasion and metastasis. Curr. Protein Pept. Sci..

[2] Zhao Z., Wang S., Barber B.L. (2014). Treatment Patterns in Patients with Metastatic Melanoma: A Retrospective Analysis. J. Skin Cancer.

[3] Agarwala S.S., Eggermont A.M.M., O’Day S., Zager J.S. (2014). Metastatic melanoma to the liver: A contemporary and comprehensive review of surgical, systemic, and regional therapeutic options. Cancer.

[4] Larkin J., Ascierto P.A., Dréno B., Atkinson V., Liszkay G., Maio M., Mandalà M., Demidov L., Stroyakovskiy D., Thomas L. (2014). Combined vemurafenib and cobimetinib in BRAF-mutated melanoma. N. Engl. J. Med..

[5] Kulkarni A., Al-Hraishawi H., Simhadri S., Hirshfield K.M., Chen S., Pine S., Jeyamohan C., Sokol L., Ali S., Teo M.L. (2017). BRAF fusion as a novel mechanism of acquired resistance to vemurafenib in BRAFV600E mutant melanoma. Clin. Cancer Res..

[6] Kaushik S., Pickup M.W., Weaver V.M. (2016). From transformation to metastasis: Deconstructing the extracellular matrix in breast cancer. Cancer Metastasis Rev..

[7] Leber M.F., Efferth T. (2009). Molecular principles of cancer invasion and metastasis. Int. J. Hyperth..

[8] Sarna M., Zadlo A., Hermanowicz P., Madeja Z., Burda K., Sarna T. (2014). Cell elasticity is an important indicator of the metastatic phenotype of melanoma cells. Exp. Dermatol..

[9] Sarna M., Krzykawska-Serda M., Jakubowska M., Zadlo A., Urbanska K. (2019). Melanin presence inhibits melanoma cell spread in mice in a unique mechanical fashion. Sci. Rep..

[10] Guenzle J., Wolf L.J., Garrelfs N.W.C., Goeldner J.M., Osterberg N., Schindler C.R., Saavedra J.E., Weyerbrock A. (2017). ATF3 reduces migration capacity by regulation of matrix metalloproteinases via NFκB and STAT3 inhibition in glioblastoma. Cell Death Discov..

[11] Lu P., Takai K., Weaver V.M., Werb Z. (2011). Extracellular Matrix Degradation and Remodeling in Development and Disease. Cold Spring Harb. Perspect. Biol..

[12] Jacob A., Prekeris R. (2015). The regulation of MMP targeting to invadopodia during cancer metastasis. Front. Cell Dev. Biol..

[13] Jiang F., Chen L., Yang Y.C., Wang X.M., Wang R.Y., Li L., Wen W., Chang Y.X., Chen C.Y., Tang J. (2015). CYP3A5 functions as a tumor suppressor in hepatocellular carcinoma by regulating mTORC2/Akt signaling. Cancer Res..

[14] Liang X., Sun R., Zhao X., Zhang Y., Gu Q., Dong X., Zhang D., Sun J., Sun B. (2017). Rictor regulates the vasculogenic mimicry of melanoma via the AKT-MMP-2/9 pathway. J. Cell. Mol. Med..

[15] Schmidt K.M., Dietrich P., Hackl C., Guenzle J., Bronsert P., Wagner C., Fichtner-Feigl S., Schlitt H.J., Geissler E.K., Hellerbrand C. (2018). Inhibition of mTORC2/RICTOR Impairs Melanoma Hepatic Metastasis. Neoplasia.

[16] Sabatini D.M. (2006). mTOR and cancer: Insights into a complex relationship. Nat. Rev. Cancer.

[17] Kim S.T., Kim S.Y., Klempner S.J., Yoon J., Kim N., Ahn S., Bang H., Kim K., Park W., Park S.H. (2017). Rapamycin-insensitive companion of mTOR (RICTOR) amplification defines a subset of advanced gastric cancer and is sensitive to AZD2014-mediated mTORC1 / 2 inhibition. Ann. Oncol..

[18] Masri J., Bernath A., Martin J., Jo O.D., Vartanian R., Funk A., Gera J. (2007). mTORC2 Activity Is Elevated in Gliomas and Promotes Growth and Cell Motility via Overexpression of Rictor. Cancer Res..

[19] Saxton R.A., Sabatini D.M. (2017). mTOR Signaling in Growth, Metabolism, and Disease. Cell.

[20] Hay N. (2005). The Akt-mTOR tango and its relevance to cancer. Cancer Cell.

[21] Serra V., Kim S.Y., Baselga J., Serra V., Eichhorn P.J.A., García-garcía C., Ibrahim Y.H., Prudkin L., Scaltriti M., Pérez-garcia J. (2014). RSK3 / 4 mediate resistance to PI3K pathway inhibitors in breast cancer Find the latest version: RSK3/4 mediate resistance to PI3K pathway inhibitors in breast cancer. J. Clin. Investig..

[22] Lang S.A., Hackl C., Moser C., Fichtner-Feigl S., Koehl G.E., Schlitt H.J., Geissler E.K., Stoeltzing O. (2010). Implication of RICTOR in the mTOR inhibitor-mediated induction of insulin-like growth factor-I receptor (IGF-IR) and human epidermal growth factor receptor-2 (Her2) expression in gastrointestinal cancer cells. Biochim. Biophys. Acta Mol. Cell Res..

[23] O’Reilly K.E., Rojo F., She Q.B., Solit D., Mills G.B., Smith D., Lane H., Hofmann F., Hicklin D.J., Ludwig D.L. (2006). mTOR inhibition induces upstream receptor tyrosine kinase signaling and activates Akt. Cancer Res..

[24] Bendell J.C., Kelley R.K., Shih K.C., Grabowsky J.A., Bergsland E., Jones S., Martin T., Infante J.R., Mischel P.S., Matsutani T. (2015). A phase I dose-escalation study to assess safety, tolerability, pharmacokinetics, and preliminary efficacy of the dual mTORC1/mTORC2 kinase inhibitor CC-223 in patients with advanced solid tumors or multiple myeloma. Cancer.

[25] Mateo J., Olmos D., Dumez H., Poondru S., Samberg N.L., Barr S., Van Tornout J.M., Jie F., Sandhu S., Tan D.S. (2016). A first in man, dose-finding study of the mTORC1/mTORC2 inhibitor OSI-027 in patients with advanced solid malignancies. Br. J. Cancer.

[26] Statz C.M., Patterson S.E., Mockus S.M. (2017). mTOR Inhibitors in Castration-Resistant Prostate Cancer: A Systematic Review. Target. Oncol..

[27] Sun B., Chen L., Fu H., Guo L., Guo H., Zhang N. (2016). Upregulation of RICTOR gene transcription by the proinflammatory cytokines through NF-κB pathway contributes to the metastasis of renal cell carcinoma. Tumor Biol..

[28] Schmidt K.M., Hellerbrand C., Ruemmele P., Michalski C.W., Kong B., Kroemer A., Hackl C., Schlitt H.J., Geissler E.K., Lang S.A. (2017). Inhibition of mTORC2 component RICTOR impairs tumor growth in pancreatic cancer models. Oncotarget.

[29] Benavides-Serrato A., Lee J., Holmes B., Landon K.A., Bashir T., Jung M.E., Lichtenstein A., Gera J. (2017). Specific blockade of Rictor-mTOR association inhibits mTORC2 activity and is cytotoxic in glioblastoma. PLoS ONE.

[30] Barrandon Y., Green H. (1987). Three clonal types of keratinocyte with different capacities for multiplication. Proc. Nati. Acad. Sci. USA.

[31] Harries M., Malvehy J., Lebbe C., Heron L., Amelio J., Szabo Z., Schadendorf D. (2016). Treatment patterns of advanced malignant melanoma (stage III–IV)—A review of current standards in Europe. Eur. J. Cancer.

[32] Forschner A., Eichner F., Amaral T., Keim U., Garbe C., Eigentler T.K. (2017). Improvement of overall survival in stage IV melanoma patients during 2011–2014: Analysis of real-world data in 441 patients of the German Central Malignant Melanoma Registry (CMMR). J. Cancer Res. Clin. Oncol..

[33] Murugan A.K. (2019). mTOR: Role in cancer, metastasis and drug resistance. Semin. Cancer Biol..

[34] Hua H., Kong Q., Zhang H., Wang J., Luo T., Jiang Y. (2019). Targeting mTOR for cancer therapy. J. Hematol. Oncol..

[35] Zhang S., Qian G., Zhang Q.Q., Yao Y., Wang D., Chen Z.G., Wang L.J., Chen M., Sun S.Y. (2019). MTORC2 suppresses GSK3-dependent snail degradation to positively regulate cancer cell invasion and metastasis. Cancer Res..

[36] Lang S.A., Moser C., Fichnter-Feigl S., Schachtschneider P., Hellerbrand C., Schmitz V., Schlitt H.J., Geissler E.K., Stoeltzing O. (2009). Targeting heat-shock protein 90 improves efficacy of rapamycin in a model of hepatocellular carcinoma in mice. Hepatology.

[37] Sarbassov D., Guertin D., Ali S., Sabatini D. (2005). Phosphorylation and regulation of Akt//PKB by the rictor-mTOR complex. Science.

[38] Chen B.W., Chen W., Liang H., Liu H., Liang C., Zhi X., Hu L.-Q., Yu X.-Z., Wei T., Ma T. (2015). Inhibition of mTORC2 Induces Cell-Cycle Arrest and Enhances the Cytotoxicity of Doxorubicin by Suppressing MDR1 Expression in HCC Cells. Mol. Cancer Ther..

[39] Geng L., Donnelly E., McMahon G., Lin P.C., Sierra-Rivera E., Oshinka H., Hallahan D. (2001). Inhibition of vascular endothelial growth factor receptor signaling leads to reversal of tumor resistance to radiotherapy. Cancer Res..

[40] Bhagwat S.V., Gokhale P.C., Crew A.P., Cooke A., Yao Y., Mantis C., Kahler J., Workman J., Bittner M., Dudkin L. (2011). Preclinical Characterization of OSI-027, a Potent and Selective Inhibitor of mTORC1 and mTORC2: Distinct from Rapamycin. Mol. Cancer Ther..

[41] Romano M.F., Avellino R., Petrella A., Bisogni R., Romano S., Venuta S. (2004). Rapamycin inhibits doxorubicin-induced NF-κB/Rel nuclear activity and enhances the apoptosis of melanoma cells. Eur. J. Cancer.

[42] Yang Z., Lei Z., Li B., Zhou Y., Zhang G.M., Feng Z.H., Zhang B., Shen G.X., Huang B. (2010). Rapamycin inhibits lung metastasis of B16 melanoma cells through down-regulating alphav integrin expression and up-regulating apoptosis signaling. Cancer Sci..

[43] Hagiwara A., Cornu M., Cybulski N., Polak P., Betz C., Trapani F., Terracciano L., Heim M.H., Rüegg M.A., Hall M.N. (2012). Hepatic mTORC2 Activates Glycolysis and Lipogenesis through Akt, Glucokinase, and SREBP1c. Cell Metab..

[44] Driscoll D.R., Karim S.A., Sano M., Gay D.M., Jacob W., Yu J., Mizukami Y., Gopinathan A., Jodrell D.I., Evans T.R.J. (2017). mTORC2 signaling drives the development and progression of pancreatic cancer. Cancer Res..

[45] Weiler M., Blaes J., Pusch S., Sahm F., Czabanka M., Luger S., Bunse L., Solecki G., Eichwald V., Jugold M. (2014). MTOR target NDRG1 confers MGMT-dependent resistance to alkylating chemotherapy. Proc. Natl. Acad. Sci. USA.

[46] Sun J., Zhang D., Bae D.H., Sahni S., Jansson P., Zheng Y., Zhao Q., Yue F., Zheng M., Kovacevic Z. (2013). Metastasis suppressor, NDRG1, mediates its activity through signaling pathways and molecular motors. Carcinogenesis.

[47] Cheng J., Xie H.Y., Xu X., Wu J., Wei X., Su R., Zhang W., Lv Z., Zheng Shusen S., Zhou L. (2011). NDRG1 as a biomarker for metastasis, recurrence and of poor prognosis in hepatocellular carcinoma. Cancer Lett..

[48] Ridley A.J. (2003). Cell Migration: Integrating Signals from Front to Back. Science.

[49] Sun D., Zhang Y., Qi Y., Zhou X., Lv G. (2015). Prognostic significance of MMP-7 expression in colorectal cancer: A meta-analysis. Cancer Epidemiol..

[50] Zhou R., Xu L., Ye M., Liao M., Du H., Chen H. (2014). Formononetin inhibits migration and invasion of MDA-MB-231 and 4T1 breast cancer cells by suppressing MMP-2 and MMP-9 through PI3K/AKT signaling pathways. Horm. Metab. Res..

[51] Oh B.Y., Hong H.K., Lee W.Y., Cho Y.B. (2017). Animal models of colorectal cancer with liver metastasis. Cancer Lett..

[52] Koch A., Lang S.A., Wild P.J., Gantner S., Mahli A., Spanier G., Berneburg M., Müller M., Bosserhoff A.K., Hellerbrand C. (2015). Glucose transporter isoform 1 expression enhances metastasis of malignant melanoma cells. Oncotarget.

[53] Guenzle J., Garrelfs N.W.C., Goeldner J.M., Weyerbrock A. (2019). Cyclooxygenase (COX) Inhibition by Acetyl Salicylic Acid (ASA) Enhances Antitumor Effects of Nitric Oxide in Glioblastoma In Vitro. Mol. Neurobiol..

[54] Beaver C.M., Ahmed A., Masters J.R. (2014). Clonogenicity: Holoclones and Meroclones Contain Stem Cells. PLoS ONE.

[55] Contag C.H., Bachmann M.H. (2002). Advances in In Vivo Bioluminescence Imaging of Gene Expression. Annu. Rev. Biomed. Eng..

[56] Hasan M.T., Schonig K., Berger S., Graewe W., Bujard H. (2001). Long-term, noninvasive imaging of regulated gene expression in living mice. Genesis.

