# Supplementary material for: Pharmacological Inhibition of mTORC2 Reduces Migration and Metastasis in Melanoma
Source: Int J Mol Sci. 2020 Dec 22;22(1):30. doi: 10.3390/ijms22010030 (PMC7792954; doi:10.3390/ijms22010030)
Supplement: Supplementary file 1 [file ijms-22-00030-s001.pdf]

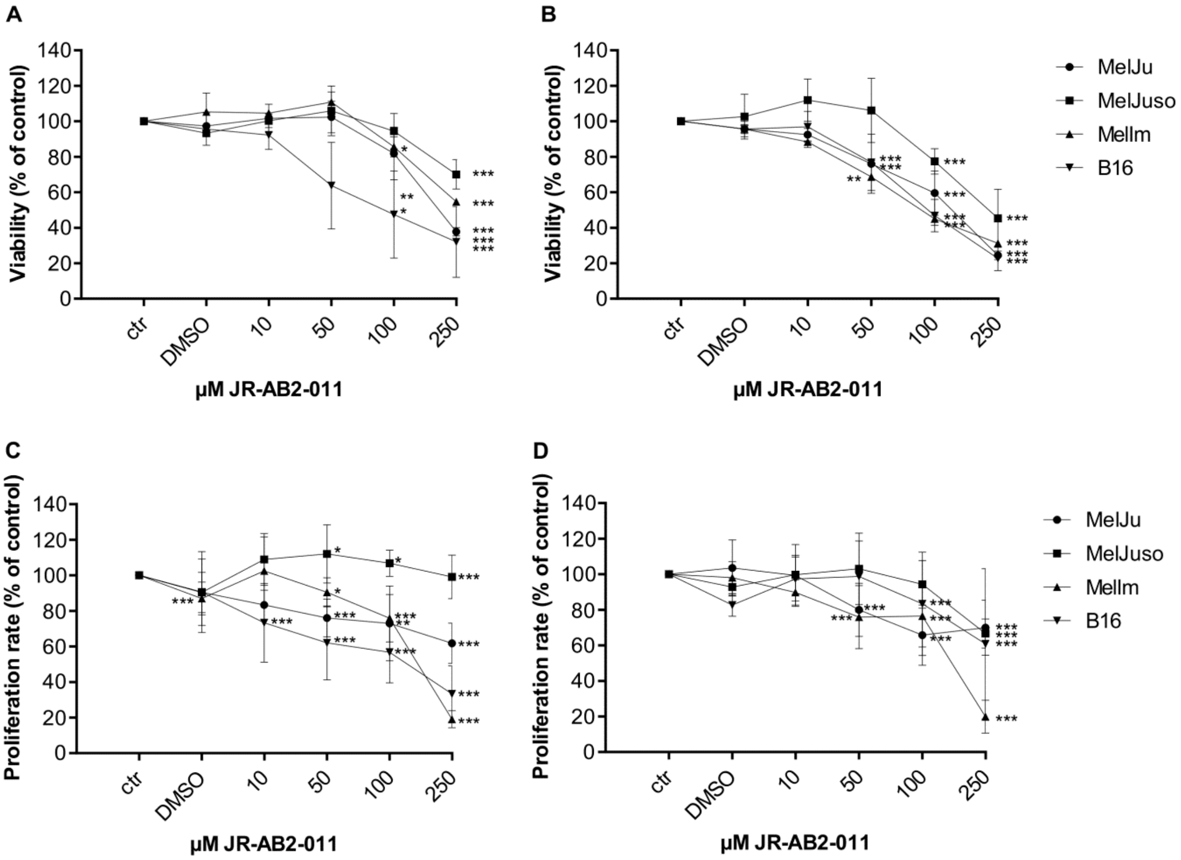

Figure S1 Viability of human and murine melanoma cells was assessed by MTT assay after treatment with JR-AB2-011 (10–250 µM) for 24 h (**A**) and 72 h (**B**). Viability was plotted relative to untreated controls set to 100% (±SD of three independent experiments). BrdU incorporation assay was performed in human and murine melanoma cells after exposure to JR-AB2-011 (10–250 µM) for 24 h (**C**) and 72 h (**D**). Treatment was normalized to untreated controls (± SD of three independent experiments). Asterisks (* $p \leq 0.05$, ** $p \leq 0.01$, *** $p \leq 0.001$) indicate significance between treatment and the control.

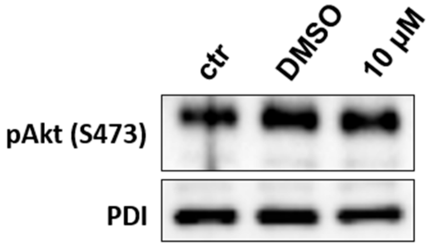

Figure S2 Representative Western Blot analysis of pAkt^{Ser473} (60 kDa) in human melanoma (MelIm) after treatment with DMSO (equivalent to 250 µM JR-AB2-011) and 10 µM JR-AB2-011 indicates no alteration in anti-apoptotic Akt signaling in melanoma cells.

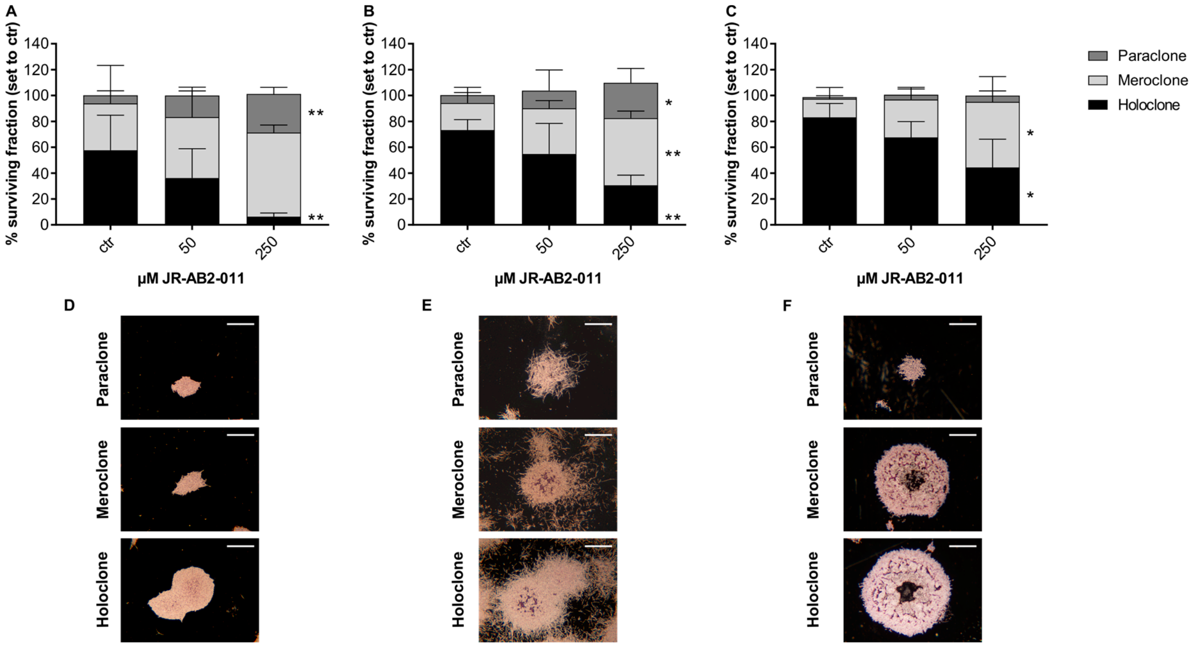

Figure S3 The morphology of the colonies of MelJuSo (**A**), MelIm (**B**) and B16 (**C**) altered significantly from the large and rapidly growing holoclone to more miscellaneous meroclones and the small and terminal paraclones in a dose-dependent manner. Representative images of MelJuSo (**D**), MelIm (**E**), and B16 (**F**) show the characteristic morphology of the para-, mero- and holoclones of each cell line. Scale bar indicates 500 µm

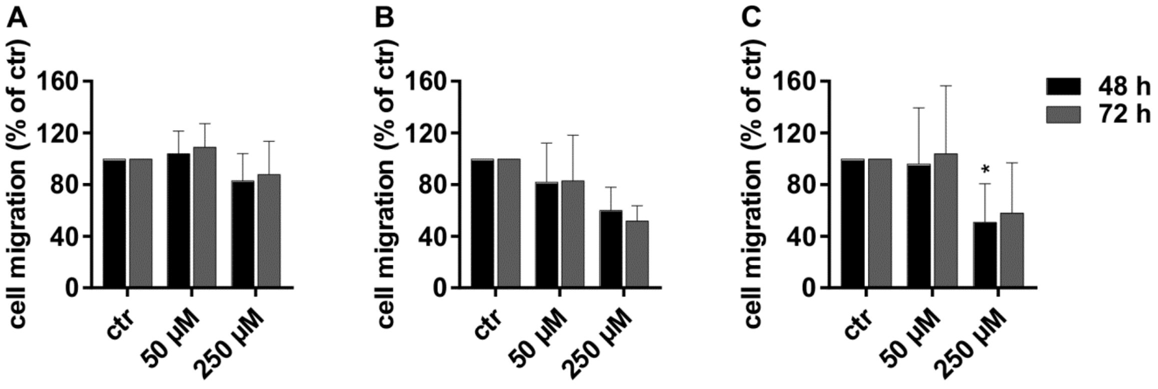

Figure S4 Real-time invasion of melanoma cells was investigated with RTCA measurement over 72 h after exposure to JR-AB2-011 (50–250 µM) of MelJu (**A**), MelIm (**B**), and B16 (**C**). Treatment was normalized to untreated controls (±SD of three independent experiments). Asterisks (* $p \leq 0.05$, ** $p \leq 0.01$, *** $p \leq 0.001$) indicate significance between treatment and the control.

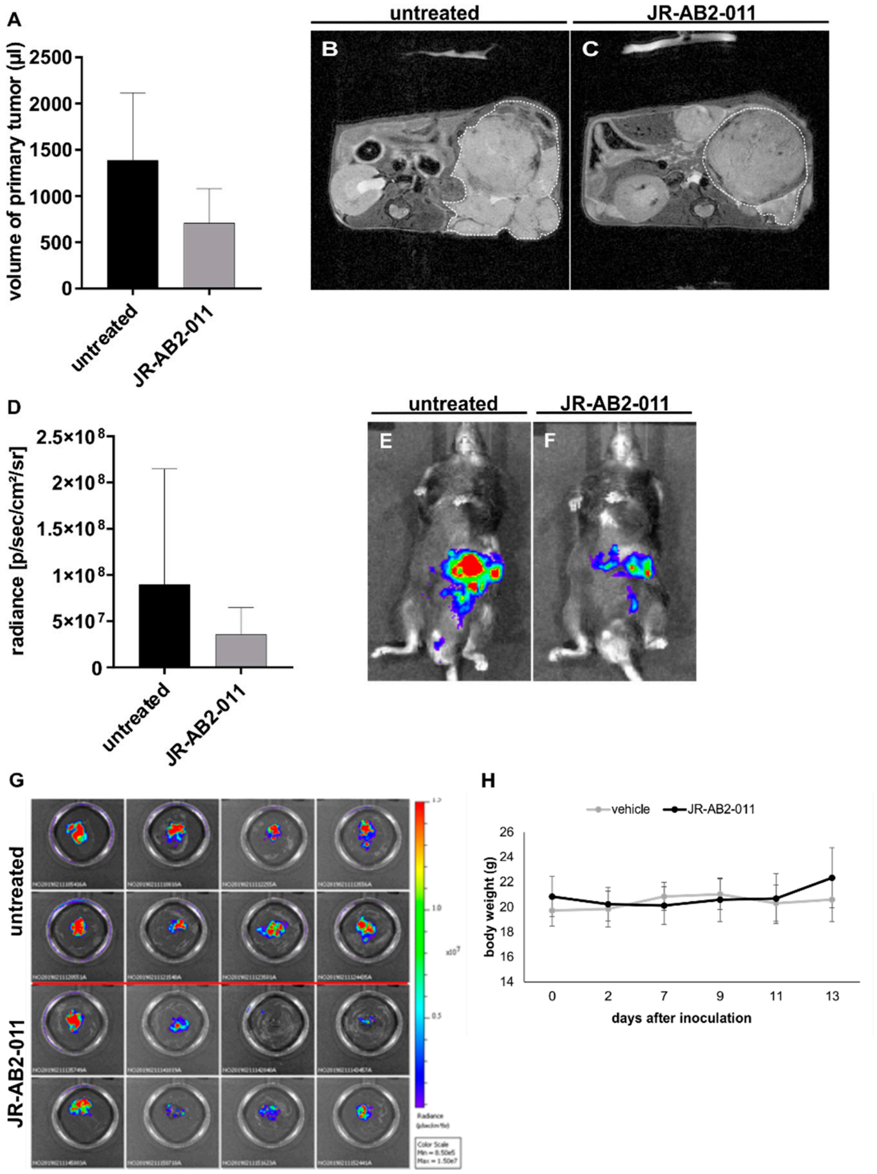

Figure S5 Investigation of MRI measurement (total amount of 30 slices per mouse, tumor volume calculated by MIPAV) revealed less tumor volume of the primary spleen tumor in the treated animal group (n = 6; 3/group) (**A**). Representative images indicate the tumor size of one single slice of untreated (**B**) and treated (**C**) mice. Due to the use of luciferase-marked B16 cells, a decrease of the total tumor burden of the living mice at day 13 was measured by bioluminescence imaging (n = 16; 8/group) (**D**). Representative images of BLI measurement show the radiance of untreated (**E**) and treated mice (**F**). Images of ex vivo liver BLI measurement of untreated (up) and treated (down) mice (n = 16; 8/group) (**G**). Course of body weight during the observation period after tumor inoculation (**H**).
